# Interdependent Polar Localization of FlhF and FlhG and Their Importance for Flagellum Formation of *Vibrio parahaemolyticus*

**DOI:** 10.3389/fmicb.2021.655239

**Published:** 2021-03-17

**Authors:** Erick Eligio Arroyo-Pérez, Simon Ringgaard

**Affiliations:** ^1^Max Planck Institute for Terrestrial Microbiology, Marburg, Germany; ^2^Department of Biology I, Microbiology, Ludwig-Maximilians-Universität München, Munich, Germany

**Keywords:** FlhG, HubP, intracellular organization, *Vibrio parahaemolyticus*, flagellum, FlhF

## Abstract

Failure of the cell to properly regulate the number and intracellular positioning of their flagella, has detrimental effects on the cells’ swimming ability. The flagellation pattern of numerous bacteria is regulated by the NTPases FlhF and FlhG. In general, FlhG controls the number of flagella produced, whereas FlhF coordinates the position of the flagella. In the human pathogen *Vibrio parahaemolyticus*, its single flagellum is positioned and formed at the old cell pole. Here, we describe the spatiotemporal localization of FlhF and FlhG in *V. parahaemolyticus* and their effect on swimming motility. Absence of either FlhF or FlhG caused a significant defect in swimming ability, resulting in absence of flagella in a Δ*flhF* mutant and an aberrant flagellated phenotype in Δ*flhG*. Both proteins localized to the cell pole in a cell cycle-dependent manner, but displayed different patterns of localization throughout the cell cycle. FlhF transitioned from a uni- to bi-polar localization, as observed in other polarly flagellated bacteria. Localization of FlhG was strictly dependent on the cell pole-determinant HubP, while polar localization of FlhF was HubP independent. Furthermore, localization of FlhF and FlhG was interdependent and required for each other’s proper intracellular localization and recruitment to the cell pole. In the absence of HubP or FlhF, FlhG forms non-polar foci in the cytoplasm of the cell, suggesting the possibility of a secondary localization site within the cell besides its recruitment to the cell poles.

## Introduction

It is essential to understand the mechanisms required for dissemination of bacteria in the environment and for many bacteria, the primary means of motion is flagella-mediated swimming motility. Correct swimming behavior heavily depends on the production of the correct number and proper placement of the flagella within the cell (Schuhmacher et al., 2015b; Blagotinsek et al., 2020; Kojima et al., 2020).

The localization of flagella in several species has been demonstrated to be mediated by landmark proteins. In particular, two proteins have been implicated in regulating the number (the ATPase FlhG) and positioning (the GTPase FlhF) of flagella in several bacterial species (Schuhmacher et al., 2015b). Interestingly, the FlhF/G system is responsible for the positioning of flagella in peritrichously, lophotrichously, and monotrichously flagellated bacteria. In some γ-proteobacteria, such as *Pseudomonas* sp., *Shewanella* sp., and *Vibrio* sp., the flagella are positioned and formed only at the old cell pole. At cell division, one daughter cell inherits these flagella at its old cell pole, whereas the second daughter is non-flagellated, but begins to produce a flagellum at its old cell pole shortly after division is finalized.

In *Vibrio alginolyticus*, *Shewanella putrefaciens*, and *P. aeruginosa*, the absence of *flhG* results in hyper-flagellated cells (Campos-García et al., 2000; Hulko et al., 2006; Kusumoto et al., 2006, 2008; Schuhmacher et al., 2015a). Hyperflagellation may be a result of increased flagellar protein production, as many flagellar genes have been shown to be upregulated in the absence of FlhG in these organisms (Dasgupta et al., 2000; Dasgupta and Ramphal, 2001; Correa et al., 2005). Deletion of *flhF* has been shown to result in swimming defects due to the absence and/or mislocalization of flagella (Pandza et al., 2000; Correa et al., 2005; Kusumoto et al., 2006; Green et al., 2009). In *Campylobacter jejuni*, *V. alginolyticus*, and *Vibrio cholerae*, the absence of *flhF* results mostly in non-flagellated non-motile cells, however, in the rare cases in which a flagellum is formed nevertheless, it is no longer positioned at the cell pole (Correa et al., 2005; Kusumoto et al., 2008; Balaban et al., 2009). A different phenotype is observed in *Pseudomonas* sp. (Pandza et al., 2000; Murray and Kazmierczak, 2006) and *S. putrefaciens* (Rossmann et al., 2015), where a single mislocalized non-polar flagellum is produced in the absence of FlhF. Importantly, FlhF is thought to establish the site of flagellum assembly by recruiting the earliest flagellar structural component FliF, which constitutes the MS-ring (Green et al., 2009; Kojima et al., 2020; Terashima et al., 2020). Fluorescence microscopy studies have shown that FlhF is localized to the bacterial cell poles in several monotrichous bacterial species, including *P. aeruginosa* (Murray and Kazmierczak, 2006), *S. putrefaciens* (Rossmann et al., 2015), *V. alginolyticus* (Kusumoto et al., 2008), and *V. cholerae* (Green et al., 2009; Yamaichi et al., 2012; Takekawa et al., 2016). In all cases, FlhF shows a specific spatiotemporal localization pattern that is cell cycle-dependent. Particularly, FlhF localizes uni-polarly to the old flagellated cell pole in young short cells and displays a bi-polar localization in older longer cells and as a consequence each daughter cell inherits FlhF localized to its old cell pole when cell division is completed (Murray and Kazmierczak, 2006; Kusumoto et al., 2008; Rossmann et al., 2015; Takekawa et al., 2016).

FlhG has been shown to negatively regulate the intracellular localization of FlhF and positively influences flagellar production by regulating FlhF GTP hydrolysis (Bange et al., 2011). In *V. alginolyticus* and *V. cholerae* species, FlhG too localizes to the cell poles (Kusumoto et al., 2008; Yamaichi et al., 2012), which fits with its function in regulating FlhF localization at this site. However, data suggests that the recruitment of FlhG to the cell pole is independent of FlhF and instead depends on interactions with the polar landmark protein HubP (Yamaichi et al., 2012).

In summary, many different bacteria use FlhF and FlhG to regulate the localization and positioning of their flagella. The system’s general function as positive and negative regulators of the flagellum synthesis, respectively, is largely conserved even among different bacterial phyla (Schuhmacher et al., 2015b; Kojima et al., 2020). Nevertheless, differences in the details of the system account for individual differences in the flagellar assembly even between members of the same genus (Schuhmacher et al., 2015b; Kojima et al., 2020). Because of these differences on the effect of the FlhF-FlhG system, also between closely related organisms, it is important to study this flagellum positioning system in different bacterial species in order to understand its importance for flagellum formation in the specific bacterium of interest.

Here, we have analyzed the importance of the FlhF-FlhG system for flagellum formation and swimming motility in the bacterium *Vibrio parahaemolyticus*. *V. parahaemolyticus* is an important human pathogen and the principal course of acute seafood-borne gastroenteritis in the world (Letchumanan et al., 2014). Furthermore, it causes substantial problems in the aquaculture industry with early mortality syndrome (EMS) of shrimps, which is an important shrimp disease particularly in Southeast Asia (Tran et al., 2013). *V. parahaemolyticus* exhibits a dimorphic life-style depending on its environmental conditions – particularly as a swimmer or a swarmer cell. Swimmer cells are monotrichously flagellated and optimized for life in liquid environments. On solid surfaces it lives as a swarmer cell, which is a cell type specialized for colonization of solid environments. Swarmer cells are highly elongated and express a distinct flagellum system in addition to the polar flagellum of swimmer cells, which results in a multiple peritrichous flagella positioned along the length of the swarmer cell (Baumann and Baumann, 1977; McCarter, 2004; Böttcher et al., 2016). In liquid environments *V. parahaemolyticus* exists as a short motile swimmer cell that is propelled by a single polar flagellum, which is positioned at the old cell pole. Swimming motility is essential for the dissemination of *V. parahaemolyticus* in the environment and for its resistance to phage attacks (Zhang et al., 2016; Freitas et al., 2020). Consequently, it is essential to study the mechanisms regulating polar flagellum formation in this specific species in order to fully understand the forces driving its spreading and survival in the environment. Here, we show that FlhF and FlhG are required for proper formation of the polar flagellum and swimming motility in *V. parahaemolyticus*. We further analyze the intracellular localization of FlhF and FlhG during the cell cycle. Both proteins localize to the bacterial cell pole in a dynamic and cell cycle-dependent manner, however, importantly their patterns of localization are distinct from each other and FlhG undergoes a different localization pattern as that of FlhF. Interestingly, their localization patterns depend on each other and in the case of FlhG also on the cell pole determinant HubP.

## Materials and Methods

### Strains and Growth Media

All strains were grown in LB medium at 37°C. When needed, indicated antibiotics were added. Genetic modifications in *V. parahaemolyticus* RIMD 2210633 were performed using standard allele exchange methods with plasmids derived from pDM4 (Milton et al., 1996). All *V. parahaemolyticus* strains were generated in a Δ*lafA* background to eliminate any cellular movement through the lateral flagella system of *V. parahaemolyticus*. *Escherichia coli* DH5αλpir was used for cloning and SM10λpir for introducing plasmids into *V. parahaemolyticus* by conjugation. All strains and plasmids used are listed in Supplementary Table 1. Primers used are listed in Supplementary Table 2. A description of each plasmid is also included as Supplementary Material.

### Swimming Assay

Swimming assays were performed as described in Ringgaard et al. (2013) and Alvarado et al. (2017).

### Fluorescence Microscopy

Fluorescence microscopy was carried out essentially as described by Ringgaard et al. (2013), Heering and Ringgaard (2016), Alvarado et al. (2017), and Heering et al. (2017). Bacterial strains for fluorescence microscopy analysis were inoculated in LB medium and cultivated at 37°C and shaking to an OD_600_ = 0.5–0.6. Cells were then spotted on a pad of 1% agarose in 50% PBS + 10% LB on a microscope slide, covered with a coverslip and imaged immediately. All microscopy was performed on a Nikon Eclipse Ti inverted Andor spinning-disk confocal microscope equipped with a 100x lens and an Andor Zyla sCMOS cooled camera and an Andor FRAPPA system. Microscopy images were analyzed using ImageJ imaging software^1^ and Metamorph Offline (version 7.10.2.240, Molecular Devices). FlhF-sfGFP fusion was imaged at 400 ms exposure, and sfGFP-FlhG at 1000 ms for all backgrounds. Demographs were constructed by measuring the fluorescence intensity profiles in Fiji and processing the data in R (3.0.1, R Foundation for Statistical Computing), using a script described by Cameron et al. (2014), Alvarado et al. (2017), Heering et al. (2017), and Muraleedharan et al. (2018).

### Transmission Electron Microscopy

Cellular cultures were propagated using identical growth conditions as those used for fluorescence microscopy analysis. Cells were grown to an OD600 = 0.5–0.6. Samples were subsequently treated as described by Ringgaard et al. (2007) and spotted on a plasma-discharged carbon-coated copper grid (Plano, Cat#S162-3) and rinsed with 0.002% uranyl acetate, blotted dry with Whatman filter paper, and further dried. TEM images were obtained with a JEOL JEM-1400 Plus 120 KV transmission electron microscope at 80 kV.

### Western Blot

Whole-cell extracts from the same cultures as used for microscopy were normalized by cell density, and equal amounts were loaded on a SDS-PAGE, blotted and probed with JL-8 anti-GFP monoclonal antibody (Takara Bio Cat# 632380, RRID:AB_10013427), and detected with horse-radish-peroxidase-conjugated anti-mouse IgG antibodies (Thermo Fisher Scientific Cat# 45-000-680, RRID:AB_2721110).

### Sample Size and Statistical Analysis

For microscopy experiments, a minimum of three biological replicates were performed, with >200 cells measured per replicate. Western blots were performed with samples from the same replicates as used for the microscopy analysis. The mean values of the replicates were plotted ± standard deviation. Statistical significance was evaluated with an ANOVA test with *post hoc* Tukey’s test. Demographs were plotted using the cellProfiles R package (Cameron et al., 2014). Ten replicates of the swimming assays were performed. The statistical significance was calculated with an ANOVA with different petri dishes as blocks. All calculations were done in R (R Development Core Team, 2008).

## Results

**FIGURE 1:**
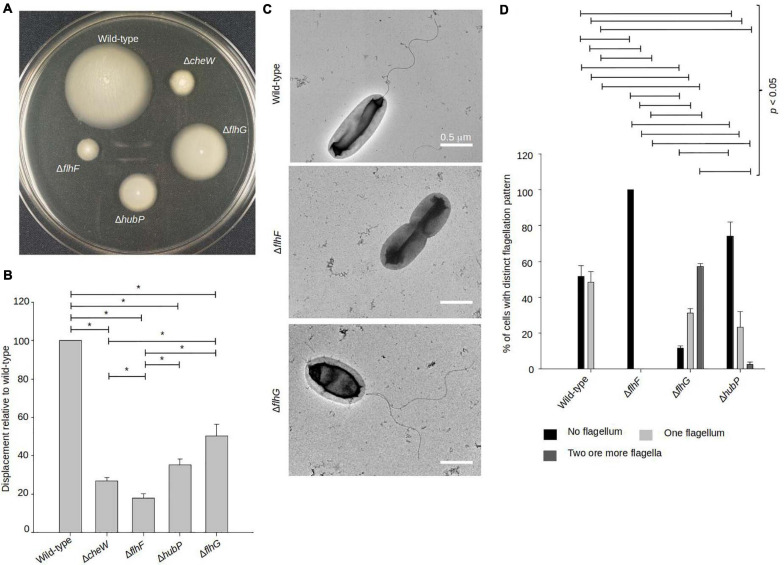
FlhF and FlhG regulate swimming and flagellum production in *V. parahaemolyticus*. **(A)** Representative image of a swimming assay in soft agar of indicated *V. parahaemolyticus* strains. **(B)** Bar graph showing the average diameter of swimming colonies of the indicated *V. parahaemolyticus* strains relative to wild-type cells. **(C)** Representative transmission electron micrographs of the indicated *V. parahaemolyticus* strains stained with uranyl acetate. **(D)** Bar graph depicting the average percentage of cells with distinct flagellation patterns, *n* = 200 cells. **(B,D)** Asterisk, *, indicates *p* < 0.05, Tested with ANOVA in blocks + Tukey HSD. Error bars indicate standard deviation.

In order to understand the importance of the FlhF-FlhG-system and HubP in motility of *V. parahaemolyticus*, we generated strains bearing in-frame deletions of either *flhF, flhG*, and *hubP* (Δ*flhF*, Δ*flhG*, and Δ*hubP*), and their effect on motility was analyzed by measuring swimming motility in soft-agar plates. As a control for no motility, we included a strain lacking the chemotaxis protein CheW (Δ*cheW*). Wild-type *V. parahaemolyticus* spread through the soft-agar, resulting in large swimming colonies, whilst no displacement was observed for the Δ*cheW* strain (Figures 1A,B). The absence of FlhF resulted in a complete lack of displacement, similar to the Δ*cheW* strain (Figures 1A,B). The absence of FlhG also significantly reduced swimming displacement by ∼50% when compared to wild-type, however, cells were still more proficient swimmers than cells lacking FlhF (Figures 1A,B). A strain lacking HubP was also significantly reduced in swimming displacement by ∼65% when compared to wild-type, however, it was significantly more swimming proficient that a strain lacking FlhF (Figures 1A,B). Interestingly, the absence of HubP resulted in a significantly stronger reduction in swimming ability than for cells lacking FlhG (Figures 1A,B).

Upon analysis of the above strains using transmission electron microscopy (TEM), it became clear that the observed swimming defects in the absence of FlhF, FlhG, or HubP was due to abnormalities in synthesis of the polar flagellum (Figures 1C,D). A polar flagellum was observed for ∼50% of wild-type cells whilst the other 50% were non-flagellated. This was in contrast to cells lacking FlhF where 100% were non-flagellated, thus showing that FlhF is required for swimming motility and flagellum production in *V. parahaemolyticus* (Figures 1C,D). A different result was obtained for cells lacking FlhG, which on the contrary is a negative regulator of flagellum synthesis, as in its absence there was a significant increase in flagellated cells, with only ∼15% of Δ*flhG* cells being non-flagellated (Figures 1C,D). Furthermore, ∼60% of Δ*flhG* cells displayed multiple flagella positioned at the same cell pole, which is virtually never seen for the wild-type. Importantly, no mislocalized non-polar flagella were observed in any case. Interestingly, the absence of *hubP* increased both the numbers of non-flagellated cells (∼70%) and multiflagellated cells (Figures 1C,D).

### FlhF and FlhG Display Distinct Cell-Cycle Dependent Polar Localization Patterns

**FIGURE 2:**
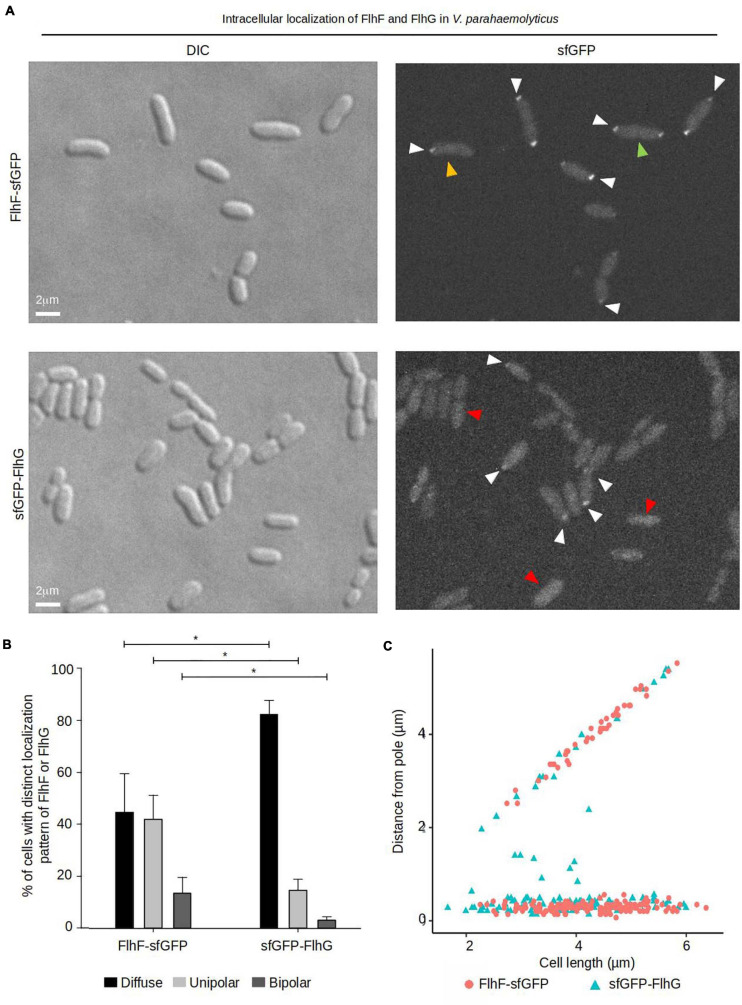
The intracellular localization of FlhF and FlhG in *V. parahaemolyticus*. **(A)** DIC and fluorescence microscopy of *V. parahaemolyticus* strains expressing FlhF-sfGFP or sfGFP-FlhG fusion proteins. White arrows indicate polar foci, orange arrows = unipolar foci, green arrows = bipolar foci, red arrow = diffuse. **(B)** Bar graph showing the percentages of cells with fluorescent foci at one, two, or no poles. Asterisk, *, indicates *p* < 0.05, tested with ANOVA + Tukey HSD. **(C)** Graph depicting the distance of FlhF-sfGFP clusters from the cell poles as a function of cell 2length.

**FIGURE 3:**
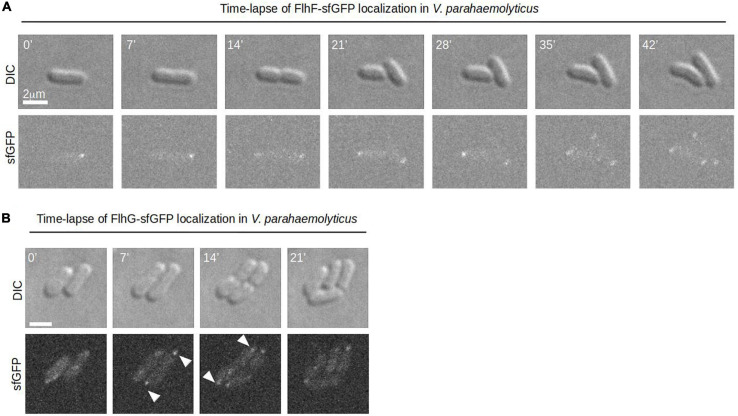
A dynamic spatiotemporal intracellular localization of FlhF and FlhG during the *V. parahaemolyticus* cell cycle. **(A,B)** Time-lapse DIC and fluorescence microscopy of *V. parahaemolyticus* strains expressing **(A)** FlhF-sfGFP or **(B)** sfGFP-FlhG fusion proteins. White numbers indicate minutes elapsed.

To further elucidate the role that FlhF and FlhG have in determining the number and position of the polar flagellum, the proteins were tagged with super folder GFP (FlhF-sfGFP and sfGFP-FlhG) and their intracellular localization visualized by fluorescence microscopy. Importantly, both fusion proteins could either completely (FlhF-sfGFP) or partially (sfGFP-FlhG) complement their respective deletion strains when the native locus was replaced by the gene encoding the fusion protein (Supplementary Figures 1A,B). This indicates that FlhF-sfGFP is fully functional while sfGFP-FlhG is at least partial functional, and thus are likely to reflect the true localization of the proteins *in vivo*. Both proteins localized in three distinct patterns: diffuse, unipolar, bipolar. Both proteins localized as discreet foci at one of the cell poles (Figure 2A, white arrows). In approximately 45% of cells FlhF was diffusely localized, whilst a significantly larger proportion (80%) of cells showed diffuse localization of FlhG (Figures 2A,B). About 60% of the cells had at least one focus of FlhF at one of the poles (Figures 2A,B), however, interestingly FlhF experienced two types of polar localization – uni-polar (∼40%) (Figures 2A,B orange arrow) and bi-polar (∼15%) (Figures 2A,B green arrow). Time-lapse microscopy showed that the two types of polar localization, was a result of a cell-cycle dependent transition in the polar localization pattern of FlhF. Particularly, time-lapse microscopy showed that in young new-borne cells FlhF localized uni-polarly at the old flagellated cell pole. Then, later in the cell cycle FlhF was recruited to the new non-flagellated cell pole, resulting in a bi-polar localization pattern. In consequence, each daughter cell inherited an FlhF cluster localized to its old pole upon completion of cell division (Figure 3A). Occasionally, we observed that FlhF became diffuse after cell division, resulting in cells with no visible foci.

Similarly, FlhG was localized at the cell poles. However, the proportion of cells with polar FlhG foci was significantly lower than that observed for FlhF. Particularly, over 80% of the population had no visible FlhG foci, which instead was localized diffusely in the cytoplasm (Figures 2A,B red arrow). When localized to the cells pole, FlhG was primarily localized in a uni-polar manner (∼18%), while only in a very small percentage (∼2%) of cells was FlhG localized in a bi-polar manner (Figures 2A–C) and observed primarily in cells very close to completing cell division. In contrast to FlhF, which was strictly localized in foci at the cell pole, FlhG foci were occassionally localized away from the cell pole within the cytoplasm of the cell (Figure 2C). We next analyzed the temporal localization pattern of FlhG during the cell-cycle using time-lapse microscopy. During the majority of the cell cycle, FlhG did not form polar foci but was instead localized diffusely in the cytoplasm (Figure 3B). Interestingly, very close to completion of cell division FlhG was recruited to both cell poles resulting in a bi-polar localization pattern and as a consequence each daughter cell inherited FlhG localized to their respective old cell poles. Soon after completion of cell division, FlhG disappeared from the cell pole and was only localized diffusely in the cytoplasm.

### FlhG Is Required for Proper Polar Localization of FlhF

**FIGURE 4:**
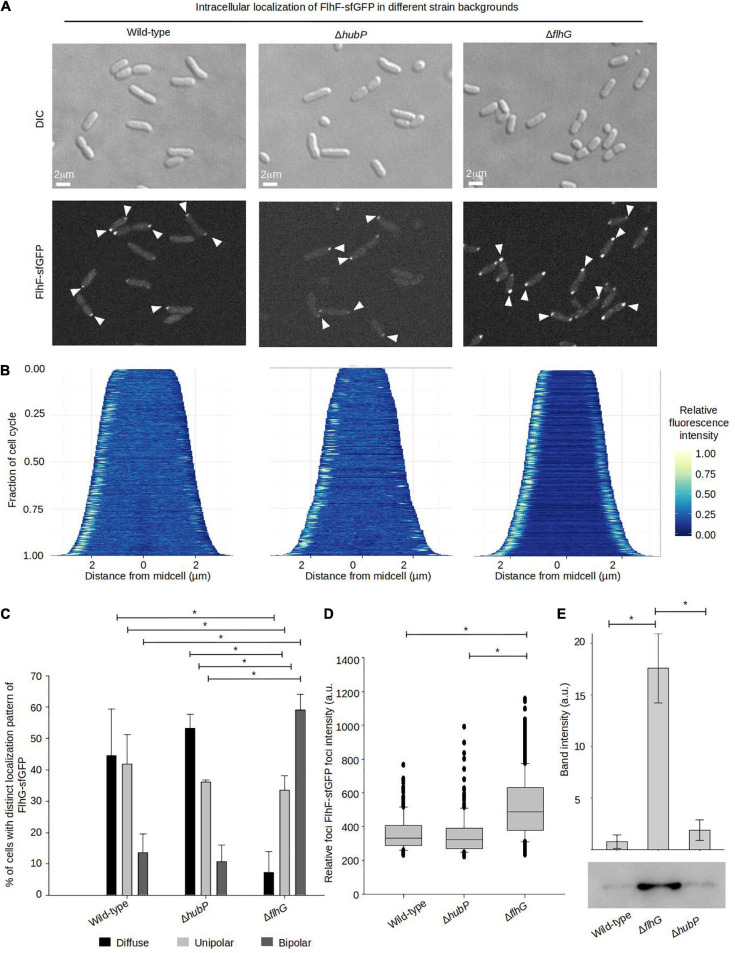
FlhG is required for proper intracellular localization of FlhF. **(A)** DIC and fluorescence microscopy of indicated *V. parahaemolyticus* strains expressing FlhF-sfGFP. White arrows indicate polar FlhF-sfGFP foci. **(B)** Demographs showing the fluorescence intensity of sfGFP along the cell length in a population of *V. parahaemolyticus* cells relative to cell length. Demographs include date from >600 cells pooled from three distinct experiments. **(C)** Bar graph showing the percentage of cells with distinct FlhF-sfGFP localization patterns in the indicated *V. parahaemolyticus* strain backgrounds. Asterisk, *, indicates *p* < 0.05, tested with ANOVA in blocks + Tukey HSD. Error bars indicate standard deviation. **(D)** Box plot showing the fluorescence intensity of polar FlhF-sfGFP foci of the indicated *V. parahaemolyticus* strains. Asterisk, *, indicates *p* < 0.05, tested with ANOVA + Tukey HSD. **(E)** Western blot with anti-GFP monoclonal antibody against whole cell extract of strains expressing FlhF-sfGFP. The bar-graph depicts the quantification of the signal detected from three biological replicates. Error bars indicate standard deviation and asterisk, *, indicates *p* < 0.05, tested with student’s *t*-test.

We next aimed to analyze how FlhF and FlhG might influence each other’s intracellular localization and the importance of HubP on their recruitment to the cell pole. To this effect, the localization of FlhF was analyzed in a Δ*hubP* and a Δ*flhG* background, respectively. FlhF was still capable of forming foci and localizing to the cell pole in the absence of HubP and no significant difference was observed in FlhF localization between wild-type and Δ*hubP* cells (Figures 4A–C). Absence of FlhG, on the other hand, had a very clear effect on the intracellular localization of FlhF localization. Particularly, there was a significant increase in the percentage of cells with polarly localized FlhF and a concomitant decrease in cells with diffusely localized FlhF, with ∼90% of cells with polarly localized FlhF in the absence of FlhG compared to ∼55% of wild-type cells (Figures 4A–C). Particularly, there was a striking increase in the number of cells with a bi-polar localization of FlhF in the absence of FlhG (∼60%) compared to wild-type (∼12%) (Figures 4A–C). Interestingly, demographic analysis showed that FlhF was recruited to the new pole earlier in the cell cycle in the absence of FlhG when compared to wild-type (Figure 4B). Furthermore, analysis of the fluorescence intensity polar FlhF clusters, showed that polar FlhF clusters were significantly brighter in a Δ*flhG* background, when compared to wild-type and Δ*hubP*, suggesting an increased level of FlhF localized to the cell pole in the absence of FlhG (Figure 4D). Consistently, Western-blot analysis determined that the level of FlhF-sfGFP was ∼8.8 fold higher in the absence of FlhG, compared to wild-type and Δ*hubP* (Figure 4E). These results, show that FlhG is required for the proper polar localization of FlhF in *V. parahaemolyticus*. They further indicate that FlhG negatively regulates the intracellular protein level of FlhF and its spatiotemporal localization and cell cycle-dependent transition from a uni-polar to a bi-polar localization pattern. Further, the results suggest that HubP has very little or no influence on the intracellular localization of FlhF.

### HubP and FlhF Are Required for Proper Recruitment of FlhG to the Cell Pole

**FIGURE 5:**
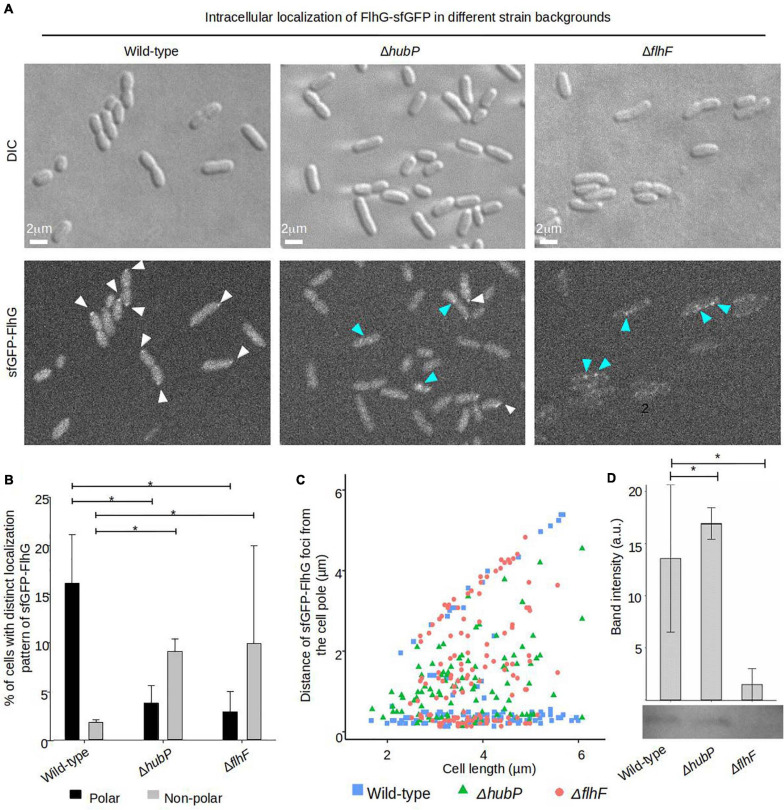
Proper intracellular localization of FlhG is regulated by HubP and FlhF. **(A)** DIC and fluorescence microscopy of sfGFP-FlhG in the indicated *V. parahaemolyticus* strains. White arrows indicate polar foci of sfGFP-FlhG and blue arrows indicate cytoplasmic clusters. **(B)** Bar graphs showing the percentage of cells with distinct localization pattern of sfGFP-FlhG in the indicated strains of *V. parahaemolyticus*. Error bars indicate standard deviation and asterisk, *, indicates *p* < 0.05, tested with ANOVA + Tukey HSD. **(C)** Graph depicting the distance of sfGFP-FlhG clusters from the cell poles as a function of cell length in the indicated *V. parahaemolyticus* strain backgrounds. **(D)** Western blot with anti-GFP monoclonal antibody against whole cell extract of strains expressing sfGFP-FlhG. The bar-graph depicts the quantification of the signal detected from three biological replicates. Error bars indicate standard deviation and asterisk, *, indicates *p* < 0.05, tested with student’s *t*-test.

Next, we analyzed the importance of FlhF and HubP on the intracellular localization of FlhG. Both FlhF and HubP were individually required for proper intracellular localization of FlhG and in the absence of either protein there was a significant reduction in the percentage of cells with polarly localized FlhG (Figures 5A,B). Where FlhG was localized as clusters in ∼15% of wild-type cells, only ∼2–3% of cells showed polarly localized FlhG in the absence of HubP or FlhF (Figures 5A,B). Interestingly, even though FlhG no longer localized as clusters at the cell pole in the absence of HubP or FlhF, it was observed to localize as distinct foci along the length of the cell in ∼10% of cells (Figures 5A–C blue arrows), while such foci only were observed in ∼2% of wild-type cells. However, our results also suggested that sfGFP-FlhG was unstable in the absence of FlhF. Particularly, after culturing of the strain, the fluorescent signal of the sfGFP-FlhG in a Δ*flhF* background, always faded in the population until it was no longer possible to detect. However, a PCR assay in all cases confirmed the gene encoding *sfGFP-flhG* in its correct locus. Western-blot analysis showed that in the Δ*flhF* background the level of sfGFP-FlhG was significantly lower than in wild-type and Δ*hubP* cells (Figure 5D). These results together suggest that both HubP and FlhF are required for the proper polar localization of FlhG and that in their absence FlhG is capable of forming non-polar clusters along the length of the cell.

## Discussion

In this study, we have investigated the spatiotemporal localization of the polar flagellum determinants FlhF and FlhG in *V. parahaemolyticus*. We showed that both FlhF and FlhG are required for proper swimming of *V. parahaemolyticus* and that absence of either protein results in a significant defect in swimming ability. Particularly, TEM analysis showed that deleting FlhF resulted in a complete absence of flagella, similar to what has been observed in *V. cholerae* and *V. alginolyticus* (Correa et al., 2005; Kusumoto et al., 2008). This shows that FlhF is essential for flagellum formation in *V. parahaemolyticus* and suggests that the function of FlhF is similar in the three *Vibrio* species (Correa et al., 2005; Kusumoto et al., 2008). Absence of FlhG in *V. parahaemolyticus* resulted in a hyperflagellation phenotype, again similar to what has been observed for other γ-proteobacteria (Correa et al., 2005; Kusumoto et al., 2008; Gao et al., 2015), suggesting that FlhG is a negative regulator of flagellum synthesis and acts to ensure that only one flagellum is formed at the cell pole in *V. parahaemolyticus*. This further supports that the FlhF-FlhG system works in very similar ways, particularly in *Vibrio* species.

We further showed that both FlhF and FlhG undergo a dynamic intracellular localization, where both proteins localized to the cell pole in a cell cycle-dependent manner. FlhF and FlhG displayed very distinct patterns of localization throughout the cell cycle. Particularly, FlhF showed a localization pattern that has been reported for FlhF in other polarly flagellated bacteria and *Vibrio* species as well (Correa et al., 2005; Murray and Kazmierczak, 2006; Rossmann et al., 2015). In young cells, FlhF was uni-polarly localized at the old flagellated cell pole. Then, later in the cell cycle FlhF was recruited to the new non-flagellated cell pole, resulting in a bi-polar localization pattern and as a result, each daughter cell inherited an FlhF cluster localized to its old cell pole. This is further supporting the conclusion that the function of FlhF is identical within *Vibrio* species and similar to that reported in other polar flagellated bacteria.

Despite the wealth of knowledge in regard to the intracellular localization of FlhF, it remains an open question how it is recruited to the cell pole. In other organisms, it has been shown that FlhG relies on the cell pole determinant protein HubP for its recruitment to the cell pole. Here, we show that FlhG in *V. parahaemolyticus* also depends on the protein HubP for its recruitment to the cell pole. But, in contrast to FlhG, the recruitment of FlhF to the pole seemed to be independent of HubP – again consistent with what has been observed for FlhF and FlhG in other *Vibrio* species (Yamaichi et al., 2012; Rossmann et al., 2015; Takekawa et al., 2016). Our data does suggest that, similar to what is observed in *V. alginolyticus* (Kusumoto et al., 2006, 2008), recruitment of FlhF to the cell pole is negatively regulated by FlhG. Particularly, in the absence of FlhG, a much larger proportion of cells showed polarly localized FlhF and there was particularly an increase in the percentage of cells with a bi-polar localization pattern of FlhF in the absence of FlhG. Additionally, FlhF foci at the cell poles were brighter in the absence of FlhG, suggesting an increased recruitment of FlhF to the cell poles in this background. In other *Vibrio* species, deleting *flhG* increases the transcription of flagellar genes, including *flhF* (Correa et al., 2005). It is likely that the increased size and number of FlhF foci observed in our *V. parahaemolyticus* strain was due to an increase in the amount of FlhF molecules present in the cell. Indeed, a Western-blot confirmed that the protein levels of FlhF-sfGFP were much higher in the Δ*flhG* strain compared to the wild-type *V. parahaemolyticus*. Furthermore, we were not able to tell if FlhG directly influences localization of FlhF at the cell poles, however, given data of the system from other organisms, which have shown that FlhG directly interacts with and regulates FlhF’s GTP hydrolysis and nucleotide bound state (Balaban et al., 2009; Bange et al., 2011; Kazmierczak and Hendrixson, 2013; Schniederberend et al., 2013), it is likely that this is also the situation in *V. parahaemolyticus*. Thus, the effect of FlhG on FlhF localization is likely a combination of its regulatory function on FlhF’s protein level within the cell and FlhG-dependent regulation of FlhF’s nucleotide cycle.

FlhG, too, has been reported to localize to the bacterial cell pole in other *Vibrio* species (Kusumoto et al., 2008; Ringgaard et al., 2011; Yamaichi et al., 2012; Rossmann et al., 2015). However, here we show that FlhG, in contrast to FlhF, remained diffusely localized in the cytoplasm for the majority of the cell cycle in *V. parahaemolyticus*, and only when the cell was close to completion of cell division was FlhG recruited to both cell poles, resulting in a bi-polar localization pattern immediately before cell division was finalized. As a result, FlhG localized to the old-cell pole of each daughter cell immediately after cell division, whereafter it was delocalized from the pole and again diffusely localized in the cytoplasm. Thus, not only did FlhF and FlhG show distinct localization patterns, but also relied on different mechanisms for their recruitment to the cell pole. Polar localization of FlhG was strictly dependent on the cell pole-determinant HubP, while polar localization of FlhF was HubP independent. This distinct dependency on HubP for their recruitment to the cell pole has been shown in other related bacterial organisms and *Vibrio* species as well (Kojima et al., 2020). This again supports the notion that FlhF and FlhG work in *V. parahaemolyticus*, in a manner similar to that reported in other *Vibrio* species. The mislocalization of FlhG from the cell pole, could be responsible for the increase in flagellation observed in the Δ*flhG* and Δ*hubP* strains. It remains to be investigated whether this effect is caused by the diminished presence of FlhG at the pole, where in wild-type it interacts with components of the flagellum assembly, or the increased presence of FlhG in the cytoplasm, where it could regulate expression of flagellar genes. A combination of both mechanisms is also possible.

Furthermore, as FlhF still localizes properly to the cell pole in the absence of HubP, where FlhG is mislocalized and found only in the cytoplasm, our data suggest that FlhG does not need to be localized to the cell pole in order to carry out its effect on the localization of FlhF. We further show that the protein levels of FlhF is similar to wild-type levels in the absence of HubP, while there is a significant increase in FlhF levels in the absence of FlhG. Altogether, these results suggest that polar localization of FlhG is not directly to regulate FlhF localization dynamics and protein levels, and thus might serve an additional purpose related to FlhG’s function in regulating proper flagellation pattern. Interestingly, we show that in the absence of either HubP or FlhF, FlhG forms non-polar foci in the cytoplasm of the cell, suggesting a secondary localization site for FlhG within the cell, besides its recruitment to the cell poles. In the absence of FlhF, there was an unstable expression of sfGFP-FlhG, which we were unable to explain. Consequently, we are not able to tell for sure if the effect on the localization of sfGFP-FlhG in the absence of FlhF, was a result of this unstable expression of the fusion protein itself or due to the lack of a direct regulatory role of FlhF on FlhG activity via FlhF-FlhG protein-protein interactions. However, as there was no change in sfGFP-FlhG expression level in the absence of HubP and FlhG formed non-polar foci in this background too, we think that these non-polar foci reflect a true secondary localization site of FlhG, which is more prevalent upon its delocalization from the cell pole. Other ParA-like ATPases are known for binding DNA. This ability is essential for their role as spatiotemporal regulators of cell components (Hester and Lutkenhaus, 2007; Ringgaard et al., 2009; Roberts et al., 2012). The FlhG homolog in *P. aeruginosa*, FleN, interacts with the master transcriptional regulator of flagella FleQ. Together they bind specific sites on the chromosome, regulating the transition between biofilm and motile lifestyles (Navarrete et al., 2019). In *V. cholerae*, both FlhF and FlhG are known transcriptional regulators of flagellar genes (Correa et al., 2005). Indeed, very recently it was shown that FlhG plays a very direct role in regulating the expression of flagellum genes in *S. putrefaciens* by connecting the initial phases of flagellum formation with the activity of the transcriptional regulator FlrA (Blagotinsek et al., 2020), which in *V. parahaemolyticus* is referred to as FlaK. It would be interesting to study whether a similar regulatory mechanism exists in *V. parahaemolyticus*. We find it noteworthy that FlhG directly interacts with a transcriptional regulator (Blagotinsek et al., 2020) and we speculate that perhaps its localization in non-polar foci, which we observe in the absence of HubP, could be related to its function in transcriptional regulation and possibly reflect an interaction with transcriptional regulators on the chromosome – hereby giving rise to the distinct focus localization sites that are particularly enhanced in the absence of HubP in *V. parahaemolyticus*. In this way, we would like to hypothesize that the localization of FlhG to the cell pole might not only reflect a function in regulating FlhF activity, but possibly to sequester it spatially to prevent its action on transcriptional regulation in the cytoplasm as a specific cell cycle check point.

Lastly, we would again like to address the polar localization of FlhF. Interestingly, our data show that in the absence of FlhG, FlhF is recruited earlier in the cell cycle to the new cell pole, resulting in an earlier establishment of its bi-polar localization. However, despite FlhF always being bi-polarly localized before cell division, and this occurring even earlier in the cell cycle in the absence of FlhG, *V. parahaemolyticus* is never flagellated at both cell poles at any point during the cell cycle – only ever at its old cell pole. This, indicates that localization of FlhF at the new cell pole is not sufficient to initiate a complete and finalized flagellum formation at this site before cell division has been completed. This further suggests that a so-far unknown factor is required for stimulation of flagellum production at the old cell pole only. Or the presence of an unknown factor prevents or inhibits FlhF function, when FlhF is positioned at the new cell pole. Thus, further studies are required in order to understand how FlhF is recruited to the cell pole and how monotrichously flagellated bacteria inhibit a flagellum to form at their new cell pole during the progression of the cell cycle, despite the flagellum determinant FlhF being bi-polarly localized for a significant part of the cell-cycle. Ultimately, knowledge of these distinct differences between species will help to shed light on the molecular details that allow bacteria to count and position their motility system in many sorts of different arrangements.

## Data Availability Statement

The raw data supporting the conclusions of this article will be made available by the authors, without undue reservation.

## Author Contributions

EEA-P carried out the experimental work. SR conceived the study. EEA-P and SR designed the research and experiments, analyzed the data, and wrote the manuscript. Both authors contributed to the article and approved the submitted version.

## Conflict of Interest

The authors declare that the research was conducted in the absence of any commercial or financial relationships that could be construed as a potential conflict of interest.

## Footnotes

1 http://rsbweb.nih.gov/ij
