# Supplementary figures and images for: Interdependent Polar Localization of FlhF and FlhG and Their Importance for Flagellum Formation of Vibrio parahaemolyticus
Source: Front Microbiol. 2021 Mar 17;12:655239. doi: 10.3389/fmicb.2021.655239 (PMC8009987; doi:10.3389/fmicb.2021.655239)

Figure S1

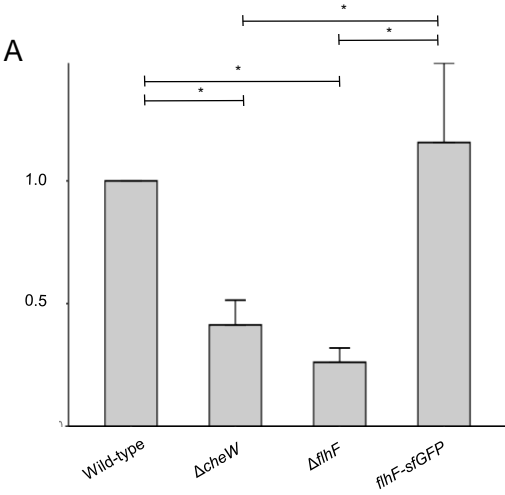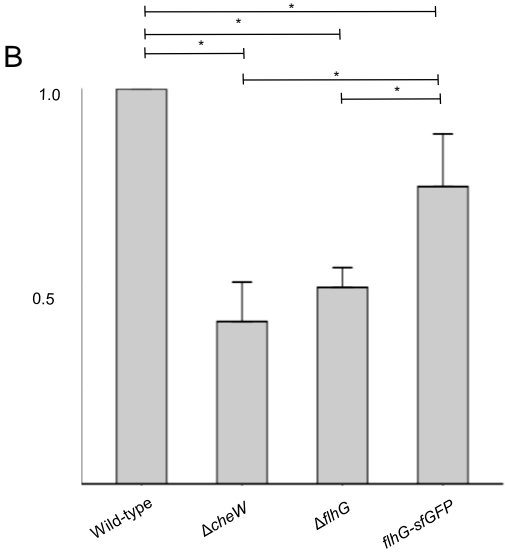

Supplement: Supplementary file 1 [file Data_Sheet_1.PDF]
