# Supplementary material for: Interdependent Polar Localization of FlhF and FlhG and Their Importance for Flagellum Formation of Vibrio parahaemolyticus
Source: Front Microbiol. 2021 Mar 17;12:655239. doi: 10.3389/fmicb.2021.655239 (PMC8009987; doi:10.3389/fmicb.2021.655239)
Supplement: Supplementary file 2 [file Table_1.DOCX]

**Supplementary materials and methods**

# Supplementary Data

**1.1 Plasmid** **construction**

**Plasmid pEP011**. The regions flanking *flhG (vp2233)* were cloned with primers del vp2233(FlhG)-a/del-flhG-b, and del-flhG-c/del vp2233(FlhG)-d, leaving the last three codons of *flhG*, which overlap with the first codons of the downstream gene *fliA,* using *V. parahaemolyticus* RIMD 2210633 chromosomal DNA as template. The resulting products were fused in a third PCR using primers del vp2233(FlhG)-a/ del vp2233(FlhG)-d. The end product was digested with XbaI and ligated in the equivalent site of vector pJH081, resulting in plasmid pEP011. The mutation in *V. parahaemolyticus* was confirmed with a PCR using primers del vp2233(FlhG)-d/del vp2233(FlhG)-chk.

**Plasmid pEP039**. Similar to plasmid pEP011, but primers SphI-sfGFP/del-vp2233(flhG)-sfGFP-b were used to amplify the upstream region of *flhG (vp2233)* from *V. parahaemolyticus* PM9 strain, and primers del-vp2233(flhG)-sfGFP-c/ del vp2233(FlhG)-d for amplification of the downstream region from *V. parahaemolyticus* RIMD 2210633. The products were fused in a third PCR using primers del SphI-sfGFP/del vp2233(FlhG)-d. The end product was digested with XbaI and SphI and ligated in the equivalent site of vector pJH081, resulting in plasmid pEP039. The mutation in *V. parahaemolyticus* was confirmed with a PCR using primers del vp2233(FlhG)-d/del vp2233(FlhG)-chk.

**Plasmid pEP040**. The region upstream of *flhG* *(vp2233)* was amplified with primers del vp2233(FlhG)-a/sfGFP-vp2233(flhG)-b from *V. parahaemolyticus* RIMD 2210633; the first 675 bp of *flhG* were amplified with primers linker-vp2233(flhG)-e/del vp2234(FlhF)-d. In addition, the gene encoding sfGFP was amplified from plasmid pJH036 using primers ins-sfGFP-cw/sfGFP-linker-d. All three PCR products were fused together in a fourth PCR with primers del vp2233(FlhG)-a/ del vp2234(FlhF)-d. The resulting product encodes sfGFP fused in frame to the N-terminus of FlhG via a 5-residue linker. This was digested with XbaI and ligated into the equivalent site of vector pJH081, resulting in plasmid pEP040. The mutation in *V. parahaemolyticus* was confirmed with a PCR using primers del vp2234(FlhF)-d/del vp2233(FlhG)-chk.

**Plasmid pEP042**. Similar to plasmid pEP040, but the region upstream of *flhF (vp2234)* was amplified using primers del vp2234(FlhF)-a/del vp2234(FlhF)-b and *V. parahaemolyticus* RIMD 2210633 chromosomal DNA. The first 700 bp of *flhG (vp2233)* were amplified with primers linker-vp2233(flhG)-e/del vp2234(FlhF)-d, and *sfGFP* was amplified from pEP040 with primers GFP-vp2233-in-del-flhF-c/sfGFP-linker-d. The resulting product encodes sfGFP fused in frame to the N-terminus of FlhG via a 5-residue linker, while omitting *flhF*. This product was digested with XbaI and ligated into the equivalent site of vector pJH081, resulting in plasmid pEP042. The mutation in *V. parahaemolyticus* was confirmed with a PCR using primers del vp2234(FlhF)-d/del vp2234(FlhF)-chk.

**Plasmid pJH003**. The regions flanking *lafA* (*vpa1548)* were cloned with primers vpa1548-del-a/vpa1548-del-b, and vpa1548-del-c/vpa1548-del-d, using *V. parahaemolyticus* RIMD 2210633 chromosomal DNA as template. The resulting products were fused in a third PCR using primers del vpa1548-del-a/vpa1548-del-d. The end product was digested with XbaI and ligated in the equivalent site of vector pDM4, resulting in plasmid pJH003. The mutation in *V. parahaemolyticus* was confirmed with a PCR using primers vpa21548-del-d/ vpa1548-del-chk.

**Plasmid pPM039.** The regions flanking *hubP* (*vp2191)* were cloned with primers vp2191-del-a/vp2191-del-b, and vp2191-del-c/vp2191-del-d, using *V. parahaemolyticus* RIMD 2210633 chromosomal DNA as template. The resulting products were fused in a third PCR using primers del vp2191-del-a/vp2191-del-d. The end product was digested with XbaI and ligated in the equivalent site of vector pDM4, resulting in plasmid pPM039. The mutation in *V. parahaemolyticus* was confirmed with a PCR using primers VP2191-del-d/ VP2191-del-chk.

**Plasmid pPM110**. The regions flanking *flhF* (*vp2234)* were cloned with primers del vp2234(FlhF)-a/del vp2234(FlhF)-b, and del vp2234(FlhF)-c/del vp2234(FlhF)-d, using *V. parahaemolyticus* RIMD 2210633 chromosomal DNA as template. The resulting products were fused in a third PCR using primers del vp2191-del-a/vp2191-del-d. The end product was digested with XbaI and ligated in the equivalent site of vector pDM4, resulting in plasmid pPM110. The mutation in *V. parahaemolyticus* was confirmed with a PCR using primers del vp2234(FlhF)-d/del vp2234(FlhF)-chk.

**Plasmid pPM188.** The last 750 bp of *flhF (vp2234)* were amplified from *V. parahaemolyticus* RIMD 2210633 with primers C-term sfGFP-vp2234-a (flhF)/C-term sfGFP-vp2234-b (flhF); the downstream region with primers C-term sfGFP-vp2234-e (flhF)/C-term sfGFP-vp2234-f (flhF), and the gene encoding sfGFP with C-term sfGFP-vp2224-c/C-term sfGFP-vp2224-d from plasmid pJH036. The three products were fused together in another PCR using primers C-term sfGFP-vp2234-a (flhF)/C-term sfGFP-vp2234-f (flhF). The obtained product, encoding the C-terminal region of FlhF fused in frame to sfGFP via a 5-residue linker, was digested with XbaI and ligated in the equivalent site of vector pDM4, resulting in plasmid pPM110. The mutation in *V. parahaemolyticus* was confirmed with a PCR using primers C-term sfGFP-vp2234-f (flhF)/del vp2233(FlhG)-chk.

# Supplementary Tables

| **Supplementary Table S1. Strains and plasmids used in this study** | | |
| --- | --- | --- |
| **Strain name** | **Genotype** | **Reference** |
| *Vibrio parahaemolyticus* RIMD 2210633 | Clinical isolate, wild type | (Makino et al., 2003) |
| *Vibrio parahaemolyticus* EP11 | *Δvp2191 (ΔhubP), Δvp2234::vp2234-sfgfp (ΔflhF::flhF-sfgfp)* | This work |
| *Vibrio parahaemolyticus* EP12 | *Δvp2234 (ΔflhF), Δvpa1548 (ΔlafA)* | This work |
| *Vibrio parahaemolyticus* EP39 | *Δvp2233 (ΔflhG)* | This work |
| *Vibrio parahaemolyticus* EP44 | *Δvp2191 (ΔhubP), Δvpa1548 (ΔlafA)* | This work |
| *Vibrio parahaemolyticus* EP45 | *Δvp2233 (ΔflhG), Δvpa1548 (ΔlafA)* | This work |
| *Vibrio parahaemolyticus* EP48 | *Δvp2233 (ΔflhG), Δvp2234::vp2234-sfgfp (ΔflhF::flhF-sfgfp)* | This work |
| *Vibrio parahaemolyticus* EP51 | *Δvp2233::sfgfp-vp2233 (ΔflhG::sfgfp-flhG)* | This work |
| *Vibrio parahaemolyticus* EP52 | *Δvp2191 (ΔhubP), Δvp2233::sfgfp-vp2233 (ΔflhG::sfgfp-flhG)* | This work |
| *Vibrio parahaemolyticus* EP53 | *Δvp2234-vp2233:: sfgfp-vp2233 (ΔflhFG::sfgfp-flhG),* | This work |
| *Vibrio parahaemolyticus* JH2 | *Δvpa1548 (ΔlafA)* | (Heering and Ringgaard, 2016) |
| *Vibrio parahaemolyticus* PM60 | *Δvp2234 (ΔflhF)* | This work |
| *Vibrio parahaemolyticus* PM69 | *Δvp2234::vp2234-sfgfp (ΔflhF::flhF-sfgfp)* | This work |
| *Vibrio parahaemolyticus* SR58 | *Δvp2225 (ΔcheW)* | (Ringgaard et al., 2013) |
| *Escherichia coli* DH5αλpir | *sup E44, ΔlacU169 (ΦlacZΔM15), recA1, endA1, hsdR17, thi-1, gyrA96, relA1*, λpir |  |
| *Escherichia coli* SM10λpir | KmR, *thi-1*, *thr, leu, tonA, lacY, supE, recA*::RP4-2-Tc::Mu, λpir |  |
| **Plasmid name** | **Relevant genotype / description** | **Reference** |
| pDM4 | Suicide vector for gene deletions | (Milton et al., 1996) |
| pJH081 | pDM4-derived suicide vector for gene deletions |  |
| pEP011 | For deletion of *flhG (vp2233)* | This work |
| pEP039 | For deletion of *flhG (vp2233)* in *flhF-sfgfp* background | This work |
| pEP040 | For insertion of *sfgfp-flhG (sfgfp-vp2233)* in the chromosome, replacing native *flhG* | This work |
| pEP042 | For insertion of *sfgfp-flhG (sfgfp-vp2233)* in a *ΔflhF* background, replacing native *flhG* | This work |
| pJH003 | For deletion of *lafA (vpa1548)* | (Heering and Ringgaard, 2016) |
| pJH036 | pBAD33 derivative encoding sfGFP | (Iyer et al., 2020) |
| pPM039 | For deletion of *hubP (vp21921)* | This work |
| pPM110 | For deletion of *flhF (vp2334)* | This work |
| pPM188 | For insertion of *flhF-sfgfp (vp2234-sfgfp)*, replacing native *flhF* | This work |

| **Supplementary Table S2. Primers used in this study** | |
| --- | --- |
| **Primer name** | **Sequence (5’ -> 3’)** |
| del vp2233(FlhG)-a | ccccc tctaga gaaggcgttgctgtcactcg |
| del vp2233(FlhG)-d | ccccc tctaga acgcactgagcttggtgcg |
| del-flhG-b | aggttatcgctttattcacagtagtccgccgccctaga |
| del-flhG-c | agggcggcggactactgtgaataaagcgataacctatgac |
| del vp2233(FlhG)-chk | gaccaaatggcatgttacatccc |
| del-vp2233(flhG)-sfGFP-b | aggttatcgctttattcac agtagtccgccgccttatttg |
| del-vp2233(flhG)-sfGFP-c | aaggcggcggactact gtgaataaagcgataacctatgac |
| SphI-sfGFP | cccc gcatgc atgagcaaaggagaagaacttttcac |
| ins-sfGFP-cw | atgagcaaaggagaagaacttttcac |
| del vp2233(FlhG)-a | ccccc tctaga gaaggcgttgctgtcactcg |
| sfGFP-vp2233(flhG)-b | tgaaaagttcttctcctttgctcatagtagtccgccgccctaga |
| sfGFP-linker-d | gagctcgaggatgtctttgtagagctcatccatgccat |
| linker-vp2233(flhG)-e | gacatcctcgagctcactgagaatatgatacacgatcaag |
| del vp2234(FlhF)-a | ccccc tctaga cgtgaagaacagcgcgagc |
| GFP-vp2233-in-del-flhF-c | cacgcatcattaatggattcatagt ggcggcggactactatgag |
| vpa1548-del-a | ccccc ctcgag tgagcgtattgctgaatttgatcc |
| vpa1548-del-b | ataaagccatcttagtctccttag |
| vpa1548-del-c | ctaaggagactaagatggctttat ggcaatgtctctacttcgttaata |
| vpa1548-del-d | ccccc ctcgag ttatgtgttccgccttcctctc |
| vpa1548-del-chk | aagtagccacatcccaaacgc |
| VP2191-del-a | ccccc tctaga agaaatgaaaatggtgtgggaaac |
| VP2191-del-b | catcaacacaagggctgctaa |
| VP2191-del-c | ttagcagcccttgtgttgatg cgccgagaagcaaaaaaactc |
| VP2191-del-d | ccccc tctaga gacaatgcgctgcacggaat |
| VP2191-del-chk | gatggaaaacggctacacca |
| del vp2234(FlhF)-b | actatgaatccattaatgatgcgtgaga |
| del vp2234(FlhF)-c | tctcacgcatcattaatggattcatagtggcggcggactactatgac |
| del vp2234(FlhF)-d | ccccc tctaga gaatacatgctacgagctcaagg |
| del vp2234(FlhF)-chk | gtttacggcatgattgatggcg |
| C-term sfGFP-vp2224-c | gacatcctcgagctc atgagcaaaggagaagaacttttcac |
| C-term sfGFP-vp2224-d | tta tttgtagagctcatccatgcc |
| C-term sfGFP-vp2234-a (flhF) | ccccc tctaga agaccaaatggcatgttacatcc |
| C-term sfGFP-vp2234-b (flhF) | gagctcgaggatgtc gagtccttcgttgtcactgttcc |
| C-term sfGFP-vp2234-e (flhF) | ggcatggatgagctctacaaa taa ggcggcggactactatgac |
| C-term sfGFP-vp2234-f (flhF) | ccccc tctaga taaagccgcaggagagcgag |

# Supplementary Figure Legends

**Supplementary Figure 1. FlhF-sfGFP and sfGFP-FlhG fusion proteins are functional for swimming behavior of *V. parahaemolyticus*. (A)** Complementation of a *Δflhf* strain with *flhF-sfGFP*. Bar graph shows the average diameter of swimming colonies of the indicated *V. parahaemolyticus* strains relative to wild-type cells. **(B)** Complementation of a *ΔflhG* strain with *sfGFP-flhG*. Bar graph shows the average diameter of swimming colonies of the indicated *V. parahaemolyticus* strains relative to wild-type cells. (A, B) Error bars indicate standard deviation. Asterisk, *, indicates p<0.05, tested with ANOVA in blocks + Tukey HSD.

# Supplementary References

Heering, J., and Ringgaard, S. (2016). Differential localization of chemotactic signaling arrays during the lifecycle of *Vibrio parahaemolyticus*. *Front. Microbiol.* 7, 1767. doi:10.3389/fmicb.2016.01767.

Iyer, S. C., Casas-Pastor, D., Kraus, D., Mann, P., Schirner, K., Glatter, T., et al. (2020). Transcriptional regulation by σ factor phosphorylation in bacteria. *Nat. Microbiol.* 5, 395–406. doi:10.1038/s41564-019-0648-6.

Makino, K., Oshima, K., Kurokawa, K., and Yokoyama, K. (2003). Genome sequence of *Vibrio parahaemolyticus*: a pathogenic mechanism distinct from that of *V. cholerae*. *Lancet* 361, 743–749.

Milton, D. L., O’Toole, R., Horstedt, P., and Wolf-Watz, H. (1996). Flagellin A is essential for the virulence of *Vibrio anguillarum*. *J. Bacteriol.* 178, 1310–9.

Ringgaard, S., Zepeda-Rivera, M., Wu, X., Schirner, K., Davis, B. M., and Waldor, M. K. (2013). ParP prevents dissociation of CheA from chemotactic signaling arrays and tethers them to a polar anchor. *Proc Natl Acad Sci U S A* 111, E255–E264. doi:10.1073/pnas.1315722111.
